# Associations of family income and healthy lifestyle with all-cause mortality

**DOI:** 10.7189/jogh.13.04150

**Published:** 2023-11-15

**Authors:** Wenbin Fang, Yawen Cao, Yingying Chen, Hengchuan Zhang, Ruyu Ni, Wan Hu, Guixia Pan

**Affiliations:** Department of Epidemiology and Biostatistics, School of Public Health, Anhui Medical University, Hefei, Anhui, China

## Abstract

**Background:**

There is a lack of evidence on whether combined lifestyle factors mediate the association between family income and all-cause mortality, as well as the joint relations between family income and lifestyle factors with mortality.

**Methods:**

Using data on family income and lifestyle factors of participants in the US National Health Interview Survey 2016-2018, we performed multivariable logistic regression models to estimate the odds ratios (ORs) and 95% confidence intervals (CI) for the association of all-cause mortality with said data.

**Results:**

We included 73 729 participants with a mean age of 47.1 years (standard deviation (SD) = 18.0), 51% of whom were women and 65% of whom were non-Hispanic Whites. There were 2284 deaths documented. After multivariable adjustment, middle-income participants had an OR of 0.73 (95% CI = 0.61-0.88) for mortality, while high-income participants had an OR of 0.47 (95% CI = 0.37-0.60) compared with low-income participants. We found that lower all-cause mortality was related to higher lifestyle scores. Adults from high-income families with lifestyle scores of 3 and 4 had an OR for mortality of 0.44 (95% CI = 0.30-0.65) compared to those from low-income families and lifestyle scores of 0 or 1. When comparing those in highest vs lowest income groups in the mediation analysis, 9.8% (95% CI = 7.4-13.0) of the relation for all-cause mortality was mediated by lifestyles. Adults from high-income families with lifestyle scores of 3 or 4 had an OR of 0.23 (95% CI = 0.17-0.33) for mortality compared with those from low-income families and lifestyle scores of 0 or 1.

**Conclusions:**

A lower risk of all-cause mortality was linked to higher family income and healthier lifestyles. Furthermore, lifestyle factors mediated a small proportion of the association between family income and mortality among US adults. Economic disparity in health may not be eliminated by changing only one’s lifestyle. Therefore, besides promoting a healthy lifestyle, we should stress how family income inequality affects health outcomes.

Over the past decade, the life expectancy of American citizens has stagnated or declined [1]. Even though most countries’ economic conditions and living standards have improved in recent years, the US has experienced increased income disparity and socioeconomic inequalities in life expectancy [2,3]. Racial and ethnic disparities in mortality were also related to socioeconomic status (SES) [4]. These differences were exacerbated during the coronavirus disease 2019 (COVID-19) outbreak, and some ethnic minority groups were at higher risk of being diagnosed with severe acute respiratory syndrome coronavirus 2 (SARS-CoV-2) [5]. As income reflects people’s current standing, it may be a more reliable indicator of SES for adults [6]. Its association with health has been extensively investigated [7-9], leading to a conclusion that higher income was associated with better health outcomes. Therefore, income inequalities must be lessened to improve public health.

Research has shown that unfavourable lifestyles are leading risk factors for chronic conditions and premature death [10-12]. A previous study found that adopting four of the five healthy lifestyle habits – never smoking cigarettes, keeping a healthy weight, performing physical activity, consuming alcohol in moderation, and eating a higher-quality diet – led to an additional ten years of life expectancy [13]. Similarly, a meta-analysis suggested that combining at least four favourable lifestyles could reduce all-cause mortality risks by 66% [14]. As an economical method for preventing chronic conditions, adopting healthy lifestyles could help with combatting the growing global burden of non-communicable diseases [15], considering that resources for global health will be insufficient if we continue to depend mainly on treatment.

Lifestyle is usually considered to be a mediator between SES and health [16]. Previous studies have shown that associations between SES or educational level and all-cause mortality were mediated by lifestyles [1,2]. Although there is an association between income and education, the two SES indicators are not interchangeable. Therefore, it remains unknown whether lifestyle mediates the association between income and mortality, and whether there is a joint relation between lifestyles and income. There is also a need to determine whether these associations remain consistent for subpopulations across various ages, sexes, races and ethnicities, educational levels, marital statuses, and chronic condition groups. To address this gap, we investigated whether combined lifestyle factors mediate the association of family income with all-cause mortality and the extent of joint relations between family income and lifestyle factors with mortality in the US population.

## METHODS

### Study population

We used the data from the 2016-2018 round of the National Health Interview Survey (NHIS) conducted on a nationally representative sample of civilian non-institutionalised US population. NHIS used a stratified, complex multistage sampling design to select families from random clusters. After that, trained interviewers randomly selected and interviewed a sample of people from these families to learn about their health status, lifestyle risk factors, and other health-related issues. The Centres for Disease Control and Prevention Institutional Review Board reviewed and approved the survey; its design, methodology, and weighting are described elsewhere [17].

We extracted data on 85 187 adults and linked their information to the National Death Index (NDI) records by 31 December 2019. We excluded those who lacked information about family income (n = 4150), lifestyle factors (n = 3401), and other covariates (n = 2877), resulting in a sample of 73 729 participants (Figure S1 in the **Online Supplementary Document**).

### Assessment of family income

NHIS investigators obtained family income information through interviews, which is presented elsewhere [18]. We used the poverty to income ratio (PIR) to measure family income, which is the ratio of family income to the federal poverty threshold. We obtained poverty thresholds for the previous calendar year from the Census Bureau and categorised the participants as having low (PIR≤1), middle (1<PIR<4), and high (PIR≥4) income [19].

### Assessment of lifestyle factors

To comprehensively assess the associations of multiple lifestyles with all-cause mortality, we defined a combined lifestyle score that included cigarette use, alcohol intake, physical activity, and body weight based on previous studies [1]. All lifestyle factors were collected using questionnaires.

We evaluated smoking status (current, daily, former, and never) according to the NHIS definitions, where never smoking was defined as smoking less than 100 cigarettes in an adult’s lifetime, which was thought to be a healthy lifestyle.

We estimated each individual’s alcohol intake using the volume and frequency of alcoholic beverages drank in questionnaires. We assumed that one drink contains 14g of alcohol [2]. We thus split the participants into five groups according to their average daily intake of pure alcohol: never drinking, former drinking, group 1 (up to 20g/d for women or 40g/d for men), group 2 (21-40g/d for women or 41-60g/d for men), and group 3 (more than 40g/d for women or 60g/d for men) [1]. We regarded moderate drinking (group 1) as the healthy standard.

As for physical activity, according to the 2018 Physical Activity Guidelines for Americans, individuals should perform 150-300 minutes of moderate-intensity physical activity (MPA), 75-150 minutes of vigorous-intensity physical activity (VPA), or an equivalent combination of both types of activity each week [20]. We multiplied the frequency and duration to determine the total MPA and VPA (minutes/week). We weighted moderate to vigorous physical activity (MVPA) by multiplying VPA by 2, per the formula MVPA (minutes/week) = VPA × 2 + MPA [20,21]. For instance, a participant who engaged 30 minutes/week of VPA and 80 minutes/week of MPA had an overall 140 minutes/week of MVPA (30 × 2 + 80 = 140 minutes/week). We separated the participants into two groups based on the total amount of MVPA: active (MVPA≥150 minutes/week) and sedentary (MVPA<150 minutes/week). Per the Physical Activity Guidelines for Americans, we regarded being physically active (MVPA≥150 minutes/week) as the health standard.

We use body mass index (BMI) to measure healthy weight. According to the World Health Organization (WHO) definition, we calculated BMI as weight (kg) divided by height (m) squared. We then classified individuals as underweight (<18.50), healthy weight (18.50-24.99), overweight (25.00-29.99), or obese (≥30.00) [22]. We defined a BMI of 18.50 to 24.99 as the favourable level.

In summary, we used the number of positive lifestyles to calculate lifestyle scores, including not smoking, drinking in moderation, engaging in the necessary exercise, and having a healthy BMI. The score of each lifestyle factor weights 0 points (unhealthy) or 1 point (healthy), and the overall score varied from 0-4, with higher scores indicating healthier lifestyles.

### Assessment of other covariates

We included the following covariates:

− Age;
− Sex;
− Race and ethnicity (non-Hispanic Black, non-Hispanic White, Hispanics, and other races);
− Marital status (married/living with a partner, divorced/separated/widowed, and never married);
− Educational attainment (low (less than a college degree), middle (some college but no bachelor’s degree), and high (bachelor’s degree or more)).

Participants self-reported information about chronic diseases, including hypertension, high cholesterol, diabetes, coronary heart disease (CHD), stroke, cancer, and chronic obstructive pulmonary disease (COPD) during the baseline assessment.

### Ascertainment of death

Individuals’ data in NHIS were linked to the NDI by 31 December 2019, as described elsewhere [23]. International Statistical Classification of Diseases and Related Health Problems, 10th Revision codes were used to identify the causes of mortality. Our primary study outcomes were all-cause mortality, with additional focus on mortality from cardiovascular disease and malignant tumors.

### Statistical analysis

We used multivariable logistic regression to produce adjusted odds ratios (ORs) relating healthy lifestyle factors and family income to all-cause mortality. We fitted three models to investigate the association of family income with mortality among all participants. In Model 1, the covariates included age, sex, race and ethnicity, marital status, and educational level. Model 2 was further adjusted for chronic diseases (hypertension, high cholesterol, CHD, stroke, COPD, cancer, and diabetes) to control the effects of comorbidities. Model 3 additionally included a lifestyle score to correct for the effects of lifestyle. Subsequently, we performed stratified analysis according to family income and lifestyle score. To increase the statistical power, we merged adults who had 0 and 1 points and those with 3 and 4 points, after which we examined whether combined lifestyle scores mediate associations of family income with mortality and the extent of joint relations between family income and lifestyle factors with mortality. Lastly, we used subgroup analysis to examine whether the results were consistent across subpopulations.

We conducted a mediation analysis to investigate whether lifestyle mediates the relationship between family income and all-cause mortality. This approach is used to examine whether the effect of the independent variable X on the dependent variable Y is mediated by the intermediate variable M, where X influences M which in turn affects Y. Using a mediation model enables the calculation of several key measures, including the average causal mediation effect (ACME), average direct effect (ADE), and total effect (TE). We calculated the proportion of ACME in TE to reflect the proportion of mediation by intermediary variable M. Our independent variable was family income, the dependent variable was all-cause mortality, and the mediating variable was lifestyle score.

We performed several sensitivity analyses. First, we excluded participants who had cancer, diabetes, and stroke at baseline to reduce residual confounding. Second, while conducting stratified analyses, we considered the lifestyle score as a continuous variable rather than merging 0-1 and 3-4 points. Finally, we performed a stratified analysis by each lifestyle factor (ie, tobacco use status, drinking status, physical activity, and BMI) to investigate the association of family income with mortality. Considering that cardiovascular disease and malignant tumors are common causes of death, we also investigated the association of family income and lifestyle with mortality due to cardiovascular disease and malignant tumors.

To assess the impact of confounding factors, we used the E-value to estimate the strength of the association between unmeasured confounders and exposure and outcome (calculated on the scale of the risk ratio) in order to explain the observed association. A high E-value indicates that a substantial amount of unmeasured confounding would be necessary to explain an effect estimate, while a low one suggests the opposite.

We adjusted weights, strata, and clusters to account for the complex multistage sampling design. Also, to reduce biases, we considered the sample weights provided on the 2019 linked mortality data. We performed all analyses in R, version 4.2.2 (R Core Team, Vienna, Austria). We considered two-sided *P* < 0.05 to be significant.

## RESULTS

### Characteristics of eligible participants

We included 73 729 participants with a mean age of 47.1 years (standard deviation (SD) = 18.0), 51% of whom were women and 65% of whom were non-Hispanic Whites (**Table 1**). We documented 2284 deaths in NHIS from 2016 to 2018, 567 from heart disease, 120 from cerebrovascular disease, and 546 from malignant tumors. Overall, adults of high family income were more likely to be men, married or living with a partner, non-Hispanic White, and better educated. Participants from high-income families were more likely to have healthy levels of tobacco smoking, physical activity, BMI, and alcohol consumption. Overall, 5.2% of the participants had no healthy lifestyle factor, while 22.8%, 34.4%, 27.9%, and 9.8% had one, two, three, or four healthy lifestyles, respectively.

**Table 1:** Baseline characteristics of participants by family income, NHIS 2016-2018*

		Family income		
	**Population (n = 73 729)**	**Low (n = 9847)**	**Middle (n = 37 301)**	**High (n = 26 581)**
**Sex**				
Female	39 710 (51.0)	6031 (58.4)	20 636 (51.8)	13 043 (47.9)
**Marital status**				
Married or living with a partner	37 607 (60.8)	2187 (33.9)	17 637 (57.1)	17 783 (73.4)
Widowed, divorced, or separated	19 368 (16.4)	3210 (23.5)	11 439 (19.2)	4719 (10.7)
Never married	16 754 (22.8)	4450 (42.6)	8225 (23.7)	4079 (15.9)
**Race/ethnicity**				
Non-Hispanic White	52 071 (65.0)	5048 (44.0)	25 565 (60.8)	21 458 (76.7)
Hispanic	8778 (15.9)	1943 (25.1)	5109 (19.5)	1726 (8.6)
Non-Hispanic Black/African American	7952 (11.7)	2036 (22.2)	4317 (12.8)	1599 (7.1)
Non-Hispanic other	4928 (7.4)	820 (8.7)	2310 (6.9)	1798 (7.6)
**Age in years, mean (SD)**	47.1 (18.0)	41.6 (18.6)	47.6 (19.1)	48.1 (16.1)
**Educational level**				
Low	25 921 (35.6)	5393 (58.1)	15 767 (43.7)	4761 (18.5)
Middle	23 115 (30.8)	3398 (31.7)	12 451 (32.8)	7266 (28.0)
High	24 693 (33.6)	1056 (10.3)	9083 (23.6)	14 554 (53.5)
**BMI**				
18.5-24.9	24 386 (33.1)	3355 (33.3)	12 003 (31.8)	9028 (34.7)
**Physical activity**				
Active	38 504 (53.2)	4155 (42.2)	17 354 (47.3)	16 995 (64.1)
**Smoking status**				
Never	44 042 (63.4)	5794 (61.9)	21 299 (61.3)	16 949(66.5)
**Alcohol consumption**				
Moderate drinking (group 1)†	47 007 (64.6)	4793 (47.2)	22 172 (60.1)	20 042 (75.4)
**Lifestyle score**				
0	4566 (5.2)	974 (8.6)	2715 (6.2)	977 (2.8)
1	17 673 (22.8)	3071 (31.4)	10 159 (26.0)	4443 (16.2)
2	25 027 (34.4)	3107 (33.2)	13 122 (36.0)	8798 (32.7)
3	19 640 (27.9)	1968 (20.1)	8795 (24.8)	8877 (34.1)
4	6823 (9.8)	727 (6.6)	2510 (7.1)	3586 (14.2)
**Self-reported chronic conditions**				
Hypertension	25 423 (30.1)	3410 (30.4)	13 858 (32.0)	8155 (27.6)
High cholesterol	22 343 (27.0)	2491 (21.9)	11 590 (27.1)	8262 (28.4)
Coronary heart disease	3980 (4.3)	553 (4.4)	2328 (5.0)	1099 (3.4)
Stroke	2708 (3.0)	561(5.0)	1611 (3.5)	536 (1.8)
COPD	3151 (3.2)	694 (5.7)	1884 (3.8)	573 (1.8)
Cancer	8233 (9.2)	743 (6.0)	4402 (9.3)	3088 (9.9)
Diabetes	8176 (9.8)	1392 (12.6)	4546 (10.9)	2238 (7.5)

SD – standard deviation, COPD – chronic obstructive pulmonary disease

*Presented as n (%) unless specified otherwise. All estimates accounted for complex survey designs.

†Group 1: Divided according to the average daily intake of pure alcohol, up to 20g/d for women or 40g/d for men.

‡Lifestyle score was defined as the number of healthy lifestyle factors, including never smoking, moderate drinking, achieving recommended physical activity, and having a healthy BMI.

### Mediation analysis

Compared with participants who had low family income, participants from high-income families had ORs of mortality of 0.38 (95% confidence interval (CI) = 0.30-0.48) when unadjusted and 0.43 (95% CI = 0.34-0.54) when adjusted for chronic diseases. After further adjusting for lifestyle score, the OR for the group was 0.47 (95% CI = 0.37-0.60) compared with participants from the low-income group.

We found that lifestyle score mediates the relation of family income with mortality. Compared with participants with low family income, the proportion mediated by the lifestyle score for all-cause mortality was 9.8% (95% CI = 7.4-13.0) for participants from high-income and 9.6% (95% CI = 6.2-17.0) for those from middle-income families (**Table 2**).

**Table 2:** Association of family income with all-cause mortality and mediation proportion of family income inequality attributed to lifestyle

	OR (95%CI)			
Family income*	**Model 1†**	**Model 2‡**	**Model 3§**	**Mediation proportion, % (95% CI)‖**
Low	1 (reference)	1 (reference)	1 (reference)	-
Middle	0.66 (0.55-0.78)	0.70 (0.59-0.84)	0.73 (0.61-0.88)	9.6 (6.2-17.0)
High	0.38 (0.30-0.48)	0.43 (0.34-0.54)	0.47 (0.37-0.60)	9.8 (7.4-13.0)

OR – odds ratio, CI – confidence interval

*Family income – low (PIR≤1), middle (1<PIR<4), and high (PIR≥4).

†Model 1: Adjusted for age, sex, race and ethnicity, marital status, and educational attainment.

‡Model 2: Model 1 + adjustment for chronic conditions (hypertension, high cholesterol, coronary heart disease, stroke, chronic obstructive pulmonary disease, cancer, and diabetes).

§Model 3: Model 2 + adjustment for lifestyle score.

‖Adjusted for age, sex, race and ethnicity, marital status, educational attainment, chronic conditions, and lifestyle score.

### Stratified and joint analysis

After adjusting for age, sex, race and ethnicity, marital status, educational level, and chronic diseases, higher lifestyle scores were associated with lower mortality regardless of family income. For instance, the ORs for mortality for participants with lifestyle scores of 3-4 compared to those with lifestyle score of 0-1 were 0.44 (95% CI = 0.30-0.65) among adults from high-income, 0.52 (95% CI = 0.40-0.69) among those from middle-income, and 0.50 (95% CI = 0.30-0.85) for those from low-income income families (**Table 3**). Similarly, independent of lifestyle score, higher family income was linked to lower all-cause mortality. For participants from high-income families, ORs for mortality were 0.60 (95% CI = 0.44-0.81), 0.33 (95% CI = 0.21-0.53), and 0.39 (95% CI = 0.20-0.72) among those with low (0-1 point), middle (2 points), and high (3-4 points) lifestyle scores, respectively, compared to those from low-income families (**Table 4**).

**Table 3:** Association of lifestyle score with all-cause mortality by family income

	Lifestyle score, OR (95% CI)†		
Family income*	0 ~ 1	2	3 ~ 4
Low	1 (reference)	0.90 (0.60-1.33)	0.50 (0.30-0.85)
E-value	NA	NA	2.18
Middle	1 (reference)	0.85 (0.72-1.00)	0.52 (0.40-0.69)
E-value	NA	NA	2.12
High	1 (reference)	0.58 (0.42-0.78)	0.44 (0.30-0.65)
E-value	NA	1.95	2.38

OR – odds ratio, CI – confidence interval, NA – not available

*Family income – low (PIR≤1), middle (1<PIR<4), and high (PIR≥4).

†Adjusted for age, sex, race and ethnicity, marital status, educational attainment, hypertension, high cholesterol, coronary heart disease, stroke, chronic obstructive pulmonary disease, cancer, and diabetes.

**Table 4:** Association of family income with all-cause mortality by lifestyle score

	Family income, OR (95% CI)*		
**Lifestyle score**	Low	Middle	High
0 ~ 1	1 (reference)	0.79 (0.63-0.99)	0.60 (0.44-0.81)
E-value	NA	1.50	1.90
2	1 (reference)	0.63 (0.44-0.92)	0.33 (0.21-0.53)
E-value	NA	1.83	2.88
3 ~ 4	1 (reference)	0.66 (0.38-1.10)	0.39 (0.21-0.72)
E-value	NA	NA	2.58

OR – odds ratio, CI – confidence interval, NA – not available

*Family income – low (PIR≤1), middle (1<PIR<4), and high (PIR≥4). Adjusted for age, sex, race and ethnicity, marital status, educational attainment, hypertension, high cholesterol, coronary heart disease, stroke, chronic obstructive pulmonary disease, cancer, and diabetes.

Individuals from high-income families with healthy lifestyles had reduced odds of mortality compared to low-family-income individuals with unhealthy lifestyles; those in the former group with lifestyle scores of 3-4 had an OR for all-cause mortality of 0.23 (95% CI = 0.17-0.33) compared to those in the latter group with lifestyle scores of 0-1 (**Table 5**).

**Table 5:** Joint association of family income and lifestyle score with all-cause mortality

	Lifestyle score, OR (95%CI)†		
Family income*	0 ~ 1	2	3 ~ 4
Low	1 (reference)	0.95 (0.63-1.44)	0.60 (0.36-0.99)
Middle	0.76 (0.61-0.94)	0.64 (0.51-0.80)	0.40 (0.29-0.55)
High	0.59 (0.44-0.78)	0.32 (0.24-0.45)	0.23 (0.17-0.33)

OR – odds ratio, CI – confidence interval

*Family income – low (PIR≤1), middle (1<PIR<4), and high (PIR≥4).

†Adjusted for age, sex, race and ethnicity, marital status, educational attainment, hypertension, high cholesterol, coronary heart disease, stroke, chronic obstructive pulmonary disease, cancer, and diabetes.

For mortality related to cardiovascular disease and malignant tumors, regardless of family income, a higher lifestyle score was associated with a decreased risk of specific-cause mortality from malignant tumors among the participants (Table S1 in the **Online Supplementary Document**). Furthermore, when stratified by lifestyle scores, we found that family income had a protective effect on specific-cause mortality related to cardiovascular disease and malignant tumors, although these associations were generally not statistically significant (Table S2 in the **Online Supplementary Document**). Finally, participants who had higher family income and adhered to better lifestyle practices exhibited a significant reduction in the risk of specific-cause mortality attributed to cardiovascular disease and malignant tumors (Table S3 in the **Online Supplementary Document**).

### Subgroup analysis

**Figure 1:**
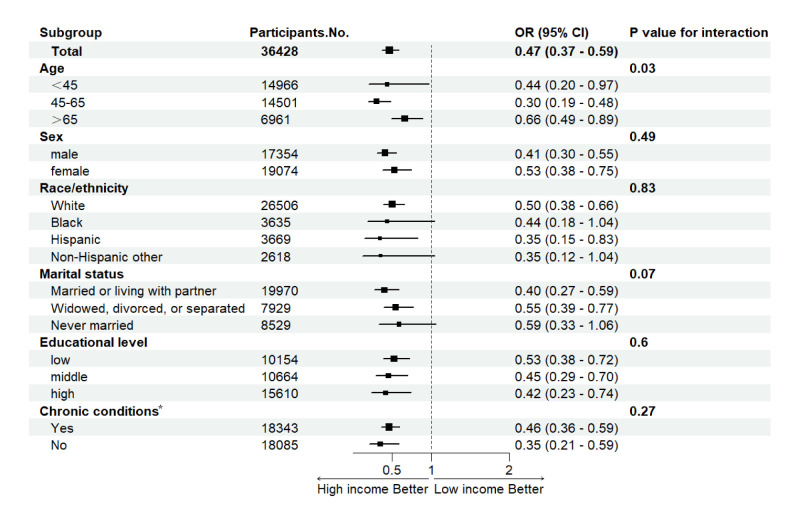
Association of family income with mortality: subgroup analyses. We mutually adjusted the model for age, sex, race and ethnicity, marital status, educational attainment, and chronic conditions, and only report on the results comparing the high with low family income. *Chronic conditions include hypertension, high cholesterol, coronary heart disease, stroke, chronic obstructive pulmonary disease, cancer, and diabetes. OR – odds ratio, CI – confidence interval. Adjusted for lifestyle score.

We observed income inequality in all-cause mortality was stronger in younger, married or living with partner, higher-educated, and individuals without chronic diseases (*P* for interaction <0.05). There was no significant association of family income with all-cause mortality among blacks, non-Hispanic other races, and never-married individuals (**Figure 1**).

**Figure 2:**
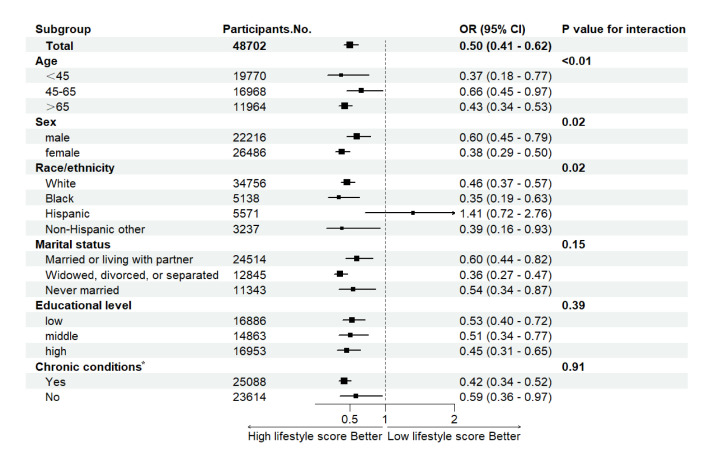
Association of lifestyle score with mortality: subgroup analyses. Adjusted for family income. We mutually adjusted the model for age, sex, race and ethnicity, marital status, educational attainment, and chronic conditions, and report only the results comparing the high (3-4 points) with the low lifestyle score (0-1 point). *Chronic conditions include hypertension, high cholesterol, coronary heart disease, stroke, chronic obstructive pulmonary disease, cancer, and diabetes. OR – odds ratio, CI – confidence interval.

For the subgroup analysis of the association of the lifestyle score with mortality, we focussed on the comparison between the low lifestyle score group (0-1 point) and the high lifestyle score group (3-4 points). Except for Hispanics, the lifestyle score was significantly associated with mortality across subgroups. It is noteworthy that there were significant disparities in the results for individuals from different ages, sex, and racial/ethnic groups (*P* for interaction <0.05) (**Figure 2**).

### Sensitivity analyses

Our findings remained consistent after excluding individuals with diabetes, cancer, or stroke at baseline (Table S4-S7 in the **Online Supplementary Document**). Notably, we found a decrease in the proportion mediated by the lifestyle score for all-cause mortality. Comparing individuals from highest and lowest income families, the proportion mediated by lifestyles was 7.9% (95% CI = 5.1-12.0) for all-cause mortality when comparing individuals from high-income to those from low-income families, and 5.6% (95% CI = 3.2-12.0) when comparing those from middle-income to those from low-income families (Table S4 in the **Online Supplementary Document**). When we considered the lifestyle score as a continuous variable, each one-point increase in lifestyle score (ie, each additional healthy lifestyle factor) among participants with high family income was associated with a 31% decrease in the odds of all-cause mortality (OR = 0.69; 95% CI = 0.60-0.78) (Table S8 in the **Online Supplementary Document**). The odds of mortality were lower for participants from high-income than those from low-income families, regardless of whether each lifestyle factor was healthy. (Table S9 in the **Online Supplementary Document**).

## DISCUSSION

For adults in our study, having a higher family income and healthier lifestyle factors was associated with a lower probability of mortality. These findings were broadly consistent among subgroups of different ages, sex, racial and ethnic, educational levels, marital statuses, and chronic conditions groups. We also determined a strong association between family income and all-cause mortality. Notably, 9.8% of the relations were mediated by lifestyles; this means that, if lifestyle level in the low-income family increased to the level of those in the high-income family, we would observe a 9.8% decrease in all-cause mortality. We further found that, compared with adults from low-income families and lifestyle scores of 0-1, those from high-income families and lifestyle scores of 3-4 had a 0.23-fold mortality risk, emphasising the reduced mortality risk associated with the combination of beneficial family income and lifestyles.

Many studies investigated economic disparity in health, with similar findings to ours. Over ten years, compared with the poorest 1% of adults, the life expectancy of the wealthiest 1% of individuals in the US increased by 14.6 years for men and 10.1 years for women [3], a phenomenon also present in other high-income countries [6,24]. Notably, although the association was strongest in adults, it started in early life [25,26]. The morbidity and mortality of specific diseases were lower for minors from higher-income families compared to those from lower-income ones [27]. This means that higher income could reduce the mortality risk, unaffected by gender and age. The social determinants of health still need to be addressed with other strategies, and promoting healthy lifestyles alone will not be sufficient to significantly reduce economic disparities in health.

Earlier studies tended to use single variables (eg, physical activity, tobacco use status, drinking status, diet, or BMI) to evaluate lifestyle. For instance, higher vigorous physical activity levels were associated with a lower risk of all-cause mortality [28]. In a dietary behavior study, cardiovascular disease mortality risk was higher when participants skipped breakfast [29]. To our knowledge, few studies have established a score to represent combined lifestyles and assess its association with mortality, and none have examined whether combined lifestyle factors mediate the association of family income with all-cause mortality. Here we found a significant inverse association between the number of healthy lifestyle factors and mortality, which is in line with previous studies [30-32]. In earlier research [33,34], physical activity (PA) was measured using metabolic equivalent (MET), yet we defined a healthy PA level as MVPA of at least 150 minutes per week. Different estimations of PA intensity would be produced by applying various definitions. According to PA guidelines [20], two minutes of MPA are equal to one minute of VPA. Therefore, we defined MVPA as assessed in minutes/week units using a combination of frequency (times/week) and duration of PA (minutes/week). This definition can also indicate a combination of different intensity PA.

We found that, among participants from high-income families, a healthy lifestyle had a stronger protective effect, and lifestyle mediated a higher proportion of the association between family income and all-cause mortality. This may have been due to the potential influence of SES on changing lifestyles. Compared to participants with higher SES, those with lower SES had lower acceptance of lifestyle changes and faced greater difficulties in changing their lifestyles. In line with these findings, Salmela et al. [35] found that women from lower socioeconomic backgrounds had a lower likelihood of recognising the importance of lifestyle advice. Furthermore, participants with lower SES were found to be less motivated to make lifestyle changes compared to those with higher SES [36-38]. Additionally, even when participating in lifestyle interventions, individuals with lower SES were more prone to dropping out [39]. Therefore, considering the significant factor of SES is essential when evaluating the influence of lifestyle on health outcomes.

Notably, individuals with lower SES often lack the ability or resources to adopt favourable lifestyles or make necessary changes to unfavourable ones, resulting in poorer lifestyle choices. This may have led us to overestimate the joint effect of family income and lifestyle. However, we performed stratified analyses according to SES and lifestyle, which showed that maintaining a healthy lifestyle significantly reduced the risk of all-cause mortality, regardless of income level. Similarly, regardless of lifestyle quality, having a higher SES also significantly reduced the risk of all-cause mortality. Therefore, both family income and lifestyles can independently influence all-cause mortality.

While both family income and lifestyle independently affect all-cause mortality, the influence of SES on lifestyle choices should not be overlooked. Accounting for socioeconomic context is crucial when designing public health interventions and policies aimed at improving health outcomes and reducing health disparities. To address these disparities and promote better health outcomes, public health interventions should consider the influence of SES on lifestyle choices. Efforts should be made to reduce barriers to adopting healthy lifestyles among individuals with lower SES. This may involve providing resources and support for lifestyle changes, increasing awareness of the importance of lifestyle modifications, and tailoring interventions to the specific needs and circumstances of different socioeconomic groups. Additionally, policies aimed at reducing socioeconomic inequalities and improving access to education, employment, and health care can have a positive impact on empowering individuals from lower SES backgrounds to make healthier lifestyle choices.

Our study has multiple strengths, including its nationally representative sample, which allows for generalisability to the general population in the US. Second, we constructed a combined healthy lifestyle score to thoroughly assess the associations between lifestyles and family income with mortality. This score could comprehensively reflect the health level of the lifestyle.

However, some limitations should be considered. First, we were unable to assess the effects of changes in lifestyles and family income during the study on all-cause mortality because the information in NHIS was collected only at baseline. Second, our assessment of SES and lifestyle was based on previously published studies, which could have introduced unknown biases. Additionally, in our defined lifestyle score, we made the potentially incorrect assumption that the effects of four lifestyle factors on all-cause mortality were equal. Third, we cannot completely eliminate the residual confounding, although we have adjusted for many potential confounders. Finally, we could not infer the causality between family income and mortality due to the study’s cross-sectional design. Therefore, our findings need to be confirmed by more longitudinal studies to show that lifestyle factors could partially explain the association between family income and mortality.

## CONCLUSIONS

We found that lifestyle factors might be a driver of income disparity in health, but also that family income significantly impacts health. Various public health programs focusing on tobacco use, exercise, and drinking are likely to have the highest health benefits when targeted at those who have low family income. Therefore, although healthy lifestyles could reduce the risk of all-cause mortality, if we only advocate healthy lifestyles without considering economic disparity, it may not significantly reduce income inequality in health. Future actions should promote healthy lifestyles to lessen the disease burden across the population, but should also focus on decreasing economic inequality in health. Future campaigns should address various socioeconomic determinants of health by putting greater effort into improving public health systems and the provision of universal health insurance.

## Additional material


Online Supplementary Document
