# Supplementary material for: Associations of family income and healthy lifestyle with all-cause mortality
Source: J Glob Health. 2023 Nov 15;13:04150. doi: 10.7189/jogh.13.04150 (PMC10644849; doi:10.7189/jogh.13.04150)
Supplement: Online Supplementary Document [file jogh-13-04150-s001.pdf]

Associations of family income and healthy lifestyle with all-cause mortality

Online Supplementary Document

Table S1. Association of lifestyle score with specific-cause mortality by family income 2

Table S2. Association of family income with specific-cause mortality by family income 3

Table S3. Joint associations of family income and lifestyle score with specific-cause mortality 4

Table S4. Association of family income with all-cause mortality and mediation proportion of family income inequality attributed to lifestyle: sensitivity analyses 5

Table S5. Association of lifestyle score with all-cause mortality by family income: sensitivity analyses..........6

Table S6. Association of family income with all-cause mortality by lifestyle score: sensitivity analyses..........7

Table S7. Adjusted odds ratios for joint associations of family income and lifestyle score with all-cause mortality: sensitivity analyses 8

Table S8. Association between lifestyle score and mortality by family income: sensitivity analyses..........9

Table S9. Association of lifestyle factors and family income with all-cause mortality: sensitivity analyses..........10

Figure S1. Inclusion and exclusion flow chart of participants...........11

Table S1. Association of lifestyle score with specific-cause mortality by family income

Family income*	Lifestyle score, OR (95% CI)†		
	0~1	2	3~4
**Cardiovascular disease**			
Low	1(Reference)	1.15 (0.56 - 2.34)	0.50 (0.17 - 1.49)
Middle	1(Reference)	0.89 (0.67 - 1.17)	0.53 (0.35 - 0.78)
High	1(Reference)	0.53 (0.33 - 0.86)	0.54 (0.31 - 0.95)
**Malignant neoplasms**			
Low	1(Reference)	0.48 (0.25 - 0.91)	0.26 (0.10 - 0.68)
Middle	1(Reference)	0.63 (0.46 - 0.85)	0.44 (0.28 - 0.69)
High	1(Reference)	0.40 (0.24 - 0.66)	0.35 (0.17 - 0.72)

OR – odds ratio, CI – confidence interval

*Family income: Low (PIR ≤ 1), Middle (1 < PIR < 4), and High (PIR ≥ 4).

†Adjusted for age, sex, race and ethnicity, marital status, educational attainment, hypertension, high cholesterol, chronic obstructive pulmonary disease, and diabetes.

Table S2. Association of family income with specific-cause mortality by family income

Lifestyle score	Family income*, OR (95% CI)†		
	Low	Middle	High
**Cardiovascular disease**			
0~1	1(Reference)	0.70 (0.46 - 1.06)	0.71 (0.42 - 1.21)
2	1(Reference)	0.48 (0.24 - 0.94)	0.33 (0.15 - 0.75)
3~4	1(Reference)	0.60 (0.21 - 1.69)	0.64 (0.21 - 1.94)
**Malignant neoplasms**			
0~1	1(Reference)	0.95 (0.65 - 1.40)	0.71 (0.44 - 1.14)
2	1(Reference)	0.95 (0.51 - 1.80)	0.45 (0.21 - 0.94)
3~4	1(Reference)	1.00 (0.37 - 2.70)	0.50 (0.17 - 1.45)

OR – odds ratio, CI – confidence interval

*Family income: Low (PIR ≤ 1), Middle (1 < PIR < 4), and High (PIR ≥ 4).

†Adjusted for age, sex, race and ethnicity, marital status, educational attainment, hypertension, high cholesterol, chronic obstructive pulmonary disease, and diabetes.

Table S3. Joint associations of family income and lifestyle score with specific-cause mortality

Family income*	Lifestyle score, OR (95% CI)†		
	0~1	2	3~4
**Cardiovascular disease**			
Low	1(Reference)	1.28 (0.60 - 2.70)	0.61 (0.22 - 1.70)
Middle	0.69 (0.46 - 1.05)	0.61 (0.39 - 0.97)	0.36 (0.21 - 0.62)
High	0.74 (0.45 - 1.22)	0.40 (0.22 - 0.71)	0.37 (0.20 - 0.72)
**Malignant neoplasms**			
Low	1(Reference)	0.54 (0.29 - 1.04)	0.31 (0.12 - 0.79)
Middle	0.87 (0.60 - 1.26)	0.55 (0.37 - 0.81)	0.39 (0.23 - 0.65)
High	0.67 (0.42 - 1.05)	0.24 (0.14 - 0.42)	0.19 (0.10 - 0.37)

OR – odds ratio, CI – confidence interval

*Family income: Low (PIR ≤ 1), Middle (1 < PIR < 4), and High (PIR ≥ 4).

†Adjusted for age, sex, race and ethnicity, marital status, educational attainment, hypertension, high cholesterol, chronic obstructive pulmonary disease, and diabetes.

Table S4. Association of family income with all-cause mortality and mediation proportion of family income inequality attributed to lifestyle: sensitivity analyses

Family income*	OR (95% CI)		Mediation proportion (%) (95% CI) §
	Model 1†	Model 2‡	
Low	1 (Reference)	1 (Reference)	-
Middle	0.61 (0.48 - 0.79)	0.64 (0.50 - 0.83)	5.6 (3.2 - 12.0)
High	0.35 (0.25 - 0.48)	0.39 (0.28 - 0.54)	7.9 (5.1 - 12.0)

OR – odds ratio, CI – confidence interval

*Family income: Low (PIR ≤ 1), Middle (1 < PIR < 4), and High (PIR ≥ 4).

†Model 1: Adjusted for age, sex, race and ethnicity, marital status, and educational attainment.

‡Model 2: Model 1 + Adjustment for lifestyle score.

§Adjusted for age, sex, race and ethnicity, marital status, educational attainment, and lifestyle score.

Table S5. Association of lifestyle score with all-cause mortality by family income: sensitivity analyses

Family income*	Lifestyle score, OR (95% CI)†		
	0~1	2	3~4
Low	1(Reference)	0.88 (0.53 - 1.50)	0.34 (0.22 - 0.70)
Middle	1(Reference)	0.89 (0.70 - 1.11)	0.65 (0.44 - 0.95)
High	1(Reference)	0.59 (0.37 - 0.90)	0.54 (0.33 - 0.90)

OR – odds ratio, CI – confidence interval

*Family income: Low (PIR ≤ 1), Middle (1 < PIR < 4), and High (PIR ≥ 4).

†Adjusted for age, sex, race and ethnicity, marital status, educational attainment, hypertension, high cholesterol, coronary heart disease, and chronic obstructive pulmonary disease.

Table S6. Association of family income with all-cause mortality by lifestyle score: sensitivity analyses

Lifestyle score	Family income*, OR (95% CI)†		
	Low	Middle	High
0~1	1(Reference)	0.67 (0.48 - 0.91)	0.47 (0.29 - 0.77)
2	1(Reference)	0.53 (0.33 - 0.86)	0.26 (0.15 - 0.46)
3~4	1(Reference)	0.82 (0.43 - 1.56)	0.47 (0.24 - 0.93)

OR – odds ratio, CI – confidence interval

*Family income: Low (PIR ≤ 1), Middle (1 < PIR < 4), and High (PIR ≥ 4).

†Adjusted for age, sex, race and ethnicity, marital status, educational attainment, hypertension, high cholesterol, coronary heart disease, and chronic obstructive pulmonary disease.

Table S7. Adjusted odds ratios for joint associations of family income and lifestyle score with all-cause mortality: sensitivity analyses

Family income*	Lifestyle score, OR (95% CI)†		
	0~1	2	3~4
Low	1 (Reference)	0.99 (0.58 - 1.69)	0.51 (0.29 - 0.90)
Middle	0.65 (0.48 - 0.88)	0.56 (0.41 - 0.77)	0.40 (0.26 - 0.63)
High	0.47 (0.30 - 0.74)	0.26 (0.17 - 0.40)	0.24 (0.15 - 0.38)

OR – odds ratio, CI – confidence interval

*Family income: Low (PIR ≤ 1), Middle (1 < PIR < 4), and High (PIR ≥ 4).

†Adjusted for age, sex, race and ethnicity, marital status, educational attainment, hypertension, high cholesterol, coronary heart disease, and chronic obstructive pulmonary disease.

Table S8. Association between lifestyle score and mortality by family income: sensitivity analyses

Family income*	lifestyle score, per 1-point increment	
	OR (95% CI)†	*p* value
low	0.80 (0.68 - 0.95)	0.01
middle	0.79 (0.73 - 0.86)	<0.001
high	0.69 (0.60 - 0.78)	<0.001

OR – odds ratio, CI – confidence interval

*Family income: Low (PIR ≤ 1), Middle (1 < PIR < 4), and High (PIR ≥ 4).

†Adjusted for age, sex, race and ethnicity, marital status, educational attainment, hypertension, high cholesterol, coronary heart disease, stroke, chronic obstructive pulmonary disease, cancer, and diabetes.

Table S9. Association of lifestyle factors and family income with all-cause mortality: sensitivity analyses

Lifestyle factors*		Family income, ORs (95% CI)†		
		Low	Middle	High
Smoking status	Healthy	1 (Reference)	0.79 (0.58 - 1.08)	0.51 (0.35 - 0.75)
	Unhealthy	1 (Reference)	0.73 (0.59 - 0.90)	0.52 (0.39 - 0.68)
Alcohol consumption	Healthy	1 (Reference)	0.67 (0.52 - 0.87)	0.38 (0.28 - 0.52)
	Unhealthy	1 (Reference)	0.78 (0.61 - 0.99)	0.64 (0.47 - 0.88)
Physical activity	Healthy	1 (Reference)	0.61 (0.42 - 0.90)	0.39 (0.25 - 0.61)
	Unhealthy	1 (Reference)	0.77 (0.63 - 0.94)	0.54 (0.41 - 0.71)
Body mass index	Healthy	1 (Reference)	0.71 (0.49 - 1.05)	0.53 (0.34 - 0.85)
	Unhealthy	1 (Reference)	0.76 (0.62 - 0.93)	0.50 (0.38 - 0.64)

OR – odds ratio, CI – confidence interval

*Never smoking, drinking in moderation (up to 20g/d for women or 40g/d for men), engaging in the prescribed amount of physical activity (≥150 min/wk), and having a healthy BMI (18.5–24.99) were regarded as healthy levels.

†All models were adjusted for age, sex, race and ethnicity, marital status, educational attainment, hypertension, high cholesterol, coronary heart disease, stroke, chronic obstructive pulmonary disease, cancer, and diabetes. Lifestyle factors were mutually adjusted.

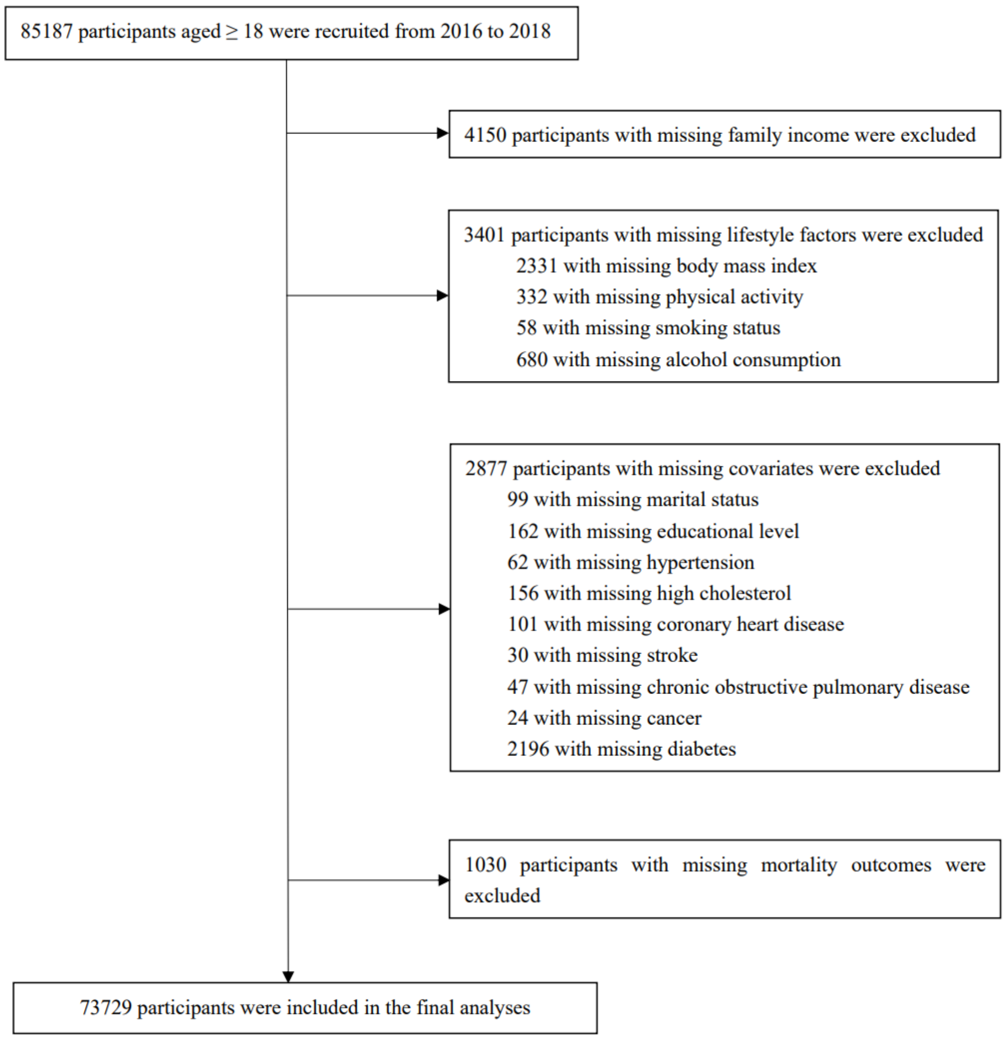

Figure S1. Inclusion and exclusion flow chart of participants.
